# The value of values-based supply chains: farmer perspective

**DOI:** 10.1007/s10460-021-10255-5

**Published:** 2021-08-23

**Authors:** Hikaru Hanawa Peterson, Gail Feenstra, Marcia Ostrom, Keiko Tanaka, Christy Anderson Brekken, Gwenael Engelskirchen

**Affiliations:** 1 Department of Applied Economics, University of Minnesota, 1994 Buford Av., St. Paul, MN 55108 USA; 2 Sustainable Agriculture Research and Education Program/Agriculture and Natural Resources, University of California, 2801 Second St., Office #262B, Davis, CA 95618 USA; 3 School of the Environment and Food Systems Program, Washington State University, 1100 N. Western Ave., Wenatchee, WA 98801 USA; 4 Department of Community and Leadership Development, University of Kentucky, Room 507, Garrigus Building, Lexington, KY 40546 USA; 5 Department of Applied Economics, Oregon State University, 240C Ballard Extension Hall, Corvallis, OR 97333 USA

**Keywords:** Values-based supply chains, Agriculture of the middle, Alternative food systems, Regional food systems, Intermediated markets, Small and mid-sized farms, Identity preserved foods

## Abstract

In the last few decades, the emergence of mid-scale, intermediated marketing channels that fall between commodity and direct markets has attracted growing interest from scholars for their potential to preserve small and mid-sized farms while scaling up alternative agrifood sourcing. When such mid-scale supply chains are formed among multiple business partners with shared ethics or values related to the qualities of the food and the business relationships along the supply chain, they may be termed “values-based supply chains (VBSCs).” Most of the research on VBSCs to date has relied primarily on a case study approach that investigates the performance of VBSCs from the perspective of VBSC founders or leaders. In contrast, this research seeks out the perspectives of farmers who participate in VBSCs. A nationwide farmer survey conducted in 2017 offers original insights on farmer motivations for participating in VBSCs and how they are being used by farmers relative to other marketing channels. We find that VBSCs serve farms of all sizes. Overall, smaller farms were more likely to market a higher percentage of overall sales through their VBSC and more likely to rank their VBSC as one of the top three marketing channels in their portfolio. But it was the larger farms that were more likely to perceive VBSC-specific benefits. Our findings confirm that while there is a limited volume of product that such regional supply chains can currently handle, farmers view VBSCs as a valuable marketing option that aligns with their own values and preserves their product’s identity.

**Supplementary Information:**

The online version contains supplementary material available at 10.1007/s10460-021-10255-5.

## Introduction

In the last few decades, food systems scholars have documented the emergence of mid-scale marketing channels in the United States that fall between commodity and direct markets (Low and Vogel 2011; Stevenson and Pirog 2008; Stevenson et al. 2011). These intermediated supply chains have been proposed as a way to fill critical gaps in regional food systems. They offer sufficiently large outlets for medium-sized producers, who may be too large or otherwise unsuited for direct markets, and offer opportunities for smaller producers to aggregate their products to reach larger markets. When these supply chains are distinguished by specific product attributes as well as shared ethics or values among participants along the chain, they have been referred to as “values-based supply chains (VBSCs).” Such strategic alliances can enable groups of farmers to aggregate their products for distribution at a larger scale while maintaining their unique business identity and receiving premiums for products differentiated by such values as quality, environment, place, or social relationships (Hardesty et al. 2014; Fleury et al. 2016).

Supported by the growing consumer demand for foods with such attributes, VBSCs have expanded. Correspondingly, these differentiated marketing channels and intermediating businesses have been increasingly positioned as the solution to the challenges faced by small and mid-sized farms in wholesale markets, sometimes referred to as the “agriculture of the middle (AOTM).” Most of the research on VBSCs has relied primarily on the case study approach, initially focused on the organizational and governance structure of these businesses and supply chain relationships, their challenges, and their defining characteristics (Lerman et al. 2012; Lerman 2012; King et al. 2013; Lev et al. 2015). Subsequently, attention has shifted toward the contribution of VBSCs to scaling up the overall quantity of foods that can be supplied through alternative agrifood channels (Fleury et al. 2016; Ostrom et al. 2017). Feenstra et al. (2011) describe the challenges and opportunities of VBSCs for institutional sales. Conner et al. (2011) describe how K-12 school districts might use VBSCs to increase regional procurement. Several researchers (Clancy and Ruhf 2010; Bloom and Hinrichs 2011; Clark and Inwood 2015) describe how VBSCs can be integrated with existing supply chain infrastructure allowing them to function as “hybrid food chains” that scale-up regional food distribution.

What remains largely unknown are farmer views on VBSCs. While it has been commonly assumed that these kinds of market innovations have emerged specifically to address the challenges of AOTM farms, little is actually known about their performance from the farmer perspective. Do these markets benefit small and medium-sized farms in distinctive ways compared to commodity and direct markets? Who participates in VBSCs? What benefits and challenges do farmers experience from participating in VBSCs? The objective of our research was to investigate differing farmer perspectives across farm types on the benefits and challenges of participating in VBSCs. Our nationwide farmer survey collected responses across a broad spectrum of established VBSCs, filling a gap in the existing literature about the contributions of VBSCs to farm sustainability and our conceptualization of the “middle” in agriculture from the farmer perspective. We examine farmers’ perceived benefits of selling to VBSCs that are both economic and sociocultural in nature, suggesting the complexity of farmers’ motivations for participating in a VBSC while simultaneously managing other marketing outlets to sustain their farm enterprise. In short, by focusing on the farmer participants in VBSCs, this paper aims to elucidate how farmers as active agents contribute to reshaping the agrifood system in accordance with their own values and management goals.

This paper consists of five sections. First, this paper discusses how our theoretical framing builds on and expands on past approaches to conceptualizing AOTM and VBSCs by drawing attention to the farmer viewpoint. In the second section, we describe our survey design and analytical approaches. Third, we present our key findings about the characteristics of farmers who participate in VBSCs and the benefits and challenges they report. Fourth, the paper returns to the discussion of the AOTM concept and analyzes the extent of the solutions posed by VBSCs for the sustainability of this sector. Finally, we conclude with an exploration of potential future work.

## Conceptualizing agriculture of the middle and values-based supply chains

In response to the US Farm Crisis of the 1980s, Browne et al. (1992) demonstrated the importance of mid-scale farms for their higher levels of productivity and efficiency in various measures compared to other farm categories. These scholars also warned about potential negative economic, environmental, and social consequences of the disappearance of a “middle” tier of mid-sized farms and marketing structures in the US agrifood system. As US agriculture has rapidly integrated into the global, industrialized agrifood system over the last four decades, the average farm size has increased and the overall numbers of income-generating small and mid-sized farms have declined (USDA ERS 2020). The 2017 Census of Agriculture indicates a continuation of these trends with only the very smallest farms (i.e., those with less than $2,500 in farm sales) and the very largest farms (those with more than $5 million in farm sales) increasing in number. Over 40% of US farms generated less than $10,000 in sales (USDA NASS 2019). This decades-long pattern of farm restructuring has hollowed out the “middle” of US agriculture—a concerning trend since historically such farms have been critical for generating household income, preserving farmland and infrastructure, sustaining rural economies, and ensuring sound stewardship of environmental resources.

In the AOTM literature, the “middle” is framed as the stratum of farms with greater reliance, or a stronger aspiration and commitment to relying on farming for a livelihood (Kirschenmann et al. 2008; Lyson et al. 2008; Stevenson et al. 2011). These “middle” farms are simultaneously an economic enterprise and a household livelihood. In balancing this duality of the farm as both an economic and sociocultural unit in society, AOTM farmers consider both economic and non-economic rationalities, logic, values, and ethics, which may be conflicting and/or competing, to make decisions about their farm operations. While multiple observable aspects including scale, business organization, type of product, and marketing strategies are used to define the “middle” sector of agriculture, Ikerd (2008) suggests that value of production, or gross farm income (GFI), may be the most salient measure of scale of farming operations. The USDA farm typology is based on gross cash farm income (GCFI), primary occupation of the operator, and ownership of the farm (Whitt et al. 2019). Farms of the middle largely correspond to the USDA’s typologies of “farming occupation farms” within “small family farms” reporting less than $350,000 GCFI, and “midsize family farms” grossing between $350,000 and $999,999. According to the 2018 Agricultural Resource Management Survey, the percentage of household income earned from farming ranged from -7% for small family farms grossing less than $150,000, to 37% for those grossing between $150,000 and $350,000, to 60% for midsize family farms (Whitt et al. 2019, p. 11). Farms grossing between $50,000 and $500,000 have been a particular focus of AOTM scholars because of their livelihood potential (Lev et al. 2015).

These AOTM farms may struggle in modern bifurcated market structures where they are too small to compete successfully in conventional commodity markets, yet too large or otherwise unsuited by location or type of product to participate in the direct marketing systems commonly associated with alternative agrifood systems. In juxtaposing the latter types of systems against globalized commodity systems, values of *sustainability*, *health*, *equity*, *sovereignty, place*, and *justice* have become increasingly salient “goals” for agrifood systems change. The geospatial proximity (e.g., local, face-to-face) and information density (e.g., EU’s geographic indications, food résumé traceability) of social interactions in the economic transactions are treated as *desirable* characteristics of *local food systems* (LFSs) and *short food supply chains* (SFSCs). While the conflation of the geospatial proximity with the relational proximity has been problematized for reproducing elitism, inequality, and hegemonic relationships within the alternative food system (DuPuis and Goodman 2005; Hinrichs 2003; Ostrom et al. 2017), the notion of “local” has been, and continues to be, a powerful framing device for mobilizing resources and spurring civic engagement to create innovative supply chain arrangements and marketing strategies. Because “local” is “socially constructed within physical, relational, moral, and discursive spaces, [its] meanings can vary vastly by product, season, geography, and the motivations and values of variously situated actors” (Ostrom et al. 2017, p. 4).

Positioned as a mid-tier marketing strategy that can potentially bolster the viability of AOTM farms, VBSCs are defined by their relational and ethical characteristics rather than their spatial characteristics (Lev et al. 2015; Stevenson et al. 2011). They are examples of creative and strategic arrangements among market actors (e.g., producers, processors, marketers) to coordinate the production and distribution of food products differentiated by the qualities associated with alternative foods, as well as the relationships along the supply chain (Lev et al. 2015). As business entities, VBSCs aggregate products from multiple farms to access larger markets at a regional scale. A few operate nationally, involving a large number of farmers and moving fairly significant sales volumes. For example, Organic Valley had projected sales of $774 million in 2012 (Stevenson 2013) and $1.1 billion in sales in 2019 (Elsen 2020). As intermediated market spaces, VBSCs are unique in their explicit use and vigorous promotion of the non-economic values associated with alternative foods such as food quality, sustainability, health, welfare, and fairness. As shown in the case studies (Lerman et al. 2012; Lerman 2012; King et al. 2013; Lev et al. 2015), these values drive the arrangement and coordination of their supply chains by using them as the key selection criteria for potential partners, including farmers and processors, as well as marketing their products to consumers. Formalized third-party certification systems, e.g., USDA Organic, sustainability certificates, and animal welfare certificates, may be employed to ensure that the integrity of these values is maintained along various points in the supply chains.

Empirical analysis of farmers’ participation in VBSCs is critical in understanding whether various types of VBSCs actually serve an agriculture of the “middle” in the US. As shown by work that uses commodity chain or system analysis methodology (e.g., Busch et al. 1991; Gereffi and Korzeniewicz 1994; Friedland 1984), supply chains are often organized very differently around various commodities. Many larger VBSCs specialize in certain categories of farm products, e.g., grains by Shepherd’s Grain, horticultural crops by Red Tomato, and dairy by Organic Valley. However, many sustainability- and locality-minded farmers in *alternative* food systems tend to diversify their farm operations, producing mixes of crops and livestock and marketing through multiple outlets, including farmers markets, CSAs (community supported agriculture), and VBSCs/food hubs (Ostrom 2017; Low et al. 2015). Furthermore, existing literature suggests that pricing mechanisms for payments to farmers vary substantially across VBSCs, as do the “premiums” obtained by various VBSC products in the marketplace. According to Lerman (2013), farmers involved in several VBSCs in Northern California do not consistently earn higher returns. Lev et al. (2015, p. 1419) similarly identifies challenges with maintaining farm identity and branding throughout the supply chain.

By using case study approaches, the past research on VBSCs has largely focused on their marketing arrangements, governance structures, and organizational innovations. Further, many of the previous case studies rely on interview data with key informants or leaders in the VBSC to infer potential contributions of VBSCs to AOTM farms. Findings from one VBSC case study may not be applicable to other VBSCs and the assessments of the lead organizers may differ significantly from those of other participants. It is unclear whether the economic benefits of VBSCs have been distributed across supply chain participants as ideally envisioned. Of particular interest is whether farmers experience economic and non-economic benefits from participating in VBSCs, which has been commonly assumed, but insufficiently interrogated.

Using a subset of our data, Brekken et al. (2019) focus on simulation scenarios showing that the average net economic impacts from VBSC participation were positive. Yet, further insight is needed to understand whether or how VBSCs help AOTM farmers balance their economic and sociocultural goals. Our findings from our national farmer survey presented in this paper confirm that the mid-sector of agriculture is multiplex and highly variable based on geography and the types of crops or livestock products produced. What extent do VBSCs positively contribute to farmers’ capacity to make a living from farming in a way that reflects their social, moral, and ethical values? The answer to this question is critical in understanding the role of VBSCs in contributing to an *alternative* agrifood system.

## Empirical approach

### Survey design and administration

To contact farmers with experience in marketing through VBSCs, we reached out to VBSC businesses with a request to share their supplier lists. We created a set of criteria for selecting the VBSCs we hoped would provide their farmer lists, including: (1) the business entity (VBSC) has value statements that are articulated in its mission statement and/or website; (2) the business entity has identifiable forward and backward supply chain linkages that go all the way back to a specific group of farms; and (3) the business entity aggregates products from multiple small and mid-sized farms. We also targeted VBSCs from varied regions across the country to include farms with a variety of crops and animal products. We invited a convenience sample of more than 30 VBSC businesses nationwide to participate, of which 19 agreed to share their supplier lists. From the combined suppliers lists, all non-farm suppliers were excluded.

The survey instrument was designed through an iterative process among the project team and farmer advisors. We created two screening questions in the beginning of the survey to be sure the farmer respondents operated a farm/ranch in 2016 and sold some portion of their products to the identified VBSC (who had provided their contact information). The survey was tailored to each VBSC, so that the 4 percent of farms on the contact list that worked with multiple VBSCs received one survey for each VBSC.

The mixed mode survey was administered by the Social and Economic Sciences Research Center (SESRC) at Washington State University. During February through May of 2017, 1954 farms were contacted through available contact methods (i.e., email, mailing address, and/or phone). After introductory contact, the SESRC followed Dillman’s Tailored Design Method (Dillman et al. 2014) that entailed a first and second mailing of the questionnaire and three reminders. The first mailing included a $5 pre-incentive. The 12-page survey was available in paper or online, in both English and Spanish (see SESRC 2017). The protocol was reviewed by the Institutional Review Boards at the authors’ universities. We received 445 responses (27.4% of those that farmed in 2016). Eliminating incomplete and non-qualified responses resulted in 298 usable responses from those who farmed and sold to a VBSC in 2016.

The farmers in this study were part of 19 VBSCs that varied widely in size and location nationwide. The number of farmer suppliers on the lists provided by the VBSCs ranged from 13 to 504, with the average being 86 farmers per VBSC. Most VBSCs only worked with farmers in their region, while four worked with farmers in two contiguous regions (USDA NASS regions, n.d.). The majority of the VBSCs participating in our study were supplied by farmers in the West, with eight supplied by farmers in the Pacific region and four in the Northwest. The median number of years the VBSCs had worked with their farmers was nearly eight years. Most of the VBSCs (84%) purchased horticultural products from farmers in the sample, while smaller percentages purchased protein, grains or oil crops: 26% eggs, 21% red meat, 21% grains, 16% oil crops, 11% poultry, 11% dairy and 5% dried beans and peas.

### Analytical methods

We are interested in how VBSCs are serving farms of various scales, particularly the AOTM farms that are grossing in the range of $50,000 to $500,000 (Agriculture of the Middle, n.d.; Lev et al. 2015). In addition, acreage is another intuitive measurement of farm size, which is useful for studying consolidation in land and crop production (MacDonald et al. 2013). We examine farms by cropland size classes that correspond to those reported in the 2017 Census of Agriculture.

Outcome variables in our study include measurements of farmers’ use of VBSCs and associated benefits and challenges that are perceived by farmers. Regarding the use of VBSCs, we consider three measurements: the proportion of output farmers sold through a VBSC, whether farmers consider their VBSC as the most important marketing outlet for their operation, and whether they consider their VBSC within the top three most important market outlets for their operation. The third measurement considers the marketing portfolio of farmers and the importance of VBSC relative to other marketing outlets.

To examine the benefits and challenges farmers perceive from their participation in their VBSC, farmers were asked in the survey to indicate the applicability of eleven items respectively—the possible benefits and challenges to their farm or ranch. Benefits can be categorized into perks from belonging to the organization, such as receiving technical assistance from the VBSC, marketing or promotional gains, such as access to new markets, and values-based benefits, such as the farmer’s environmental values being communicated to consumers. Challenges largely included transactional costs related to arrangements regarding volume or logistics, and potential practice or production standards required by the VBSCs. In addition to looking at benefits and challenges individually, factor analysis reduced the number of items to summative factors and enabled a more insightful synthesis (Cleff 2019). Factor scores were generated for farmers, who responded to at least one of the items within the benefit or cost category.

To gain insight on the varying experiences of farmers with VBSCs, the outcome variables are regressed on farm characteristics. The key farm characteristics of interest are the structural ones that define the AOTM, including size, measured by GFI and acreage, and percent of household income from farming. Other farm characteristics include commodities produced and the region where the farm is located, controlling for operator characteristics such as gender and age. Thus, our regression model can be expressed as:

$${y}_{i}=f\left({FarmSize}_{i}, {\%IncFarm}_{i},{Commodities}_{i},{Region}_{i}, {OperatorCharacteristics}_{i}\right)$$

where ${y}_{i}$ is the outcome variable for farm *i*. The standard errors are clustered by VBSCs to account for intragroup correlation. The estimation methods correspond to the nature of the outcome variables. A tobit model is used for the percent of crops sold to VBSCs; a logit model is used for binary outcome variables; and ordinary least squares is used for generated factors of benefits and challenges which are logarithmically transformed for ease of interpretation.

This study is limited by the convenience nature of the VBSCs included, as well as the number of VBSCs that participated in the study. As with all survey work, findings are subject to self-selection bias. Nonetheless, our study provides a valuable, original insight into farmers’ perspectives on VBSCs, specifically how this marketing channel is being used relative to other marketing channels and how farmers perceive the economic and sociocultural aspects.

## Results

First, we describe the characteristics of the farmers surveyed and how particular characteristics varied by GFI and by acres operated. Then, we present how farmers report the importance of VBSCs for their operations and the benefits and challenges they face in marketing through VBSCs. Lastly, we report the regression results.

### Who are VBSC participating farms and farmers?

As Table 1 shows, the greatest number of the farmers surveyed (44%) fell into the medium-scale or AOTM category ($50,000-$500,000). Another 39% fell into the large farm category (> $500,000). The remaining 17% had under $50,000 in sales. This distribution is markedly different from the 2017 Ag Census showing much higher percentages in the very small farm categories, because the Census includes retired and off-farm occupation farmers, which constitute more than half of U.S. farms (Whitt et al. 2019). The farmers in our survey were those who were already selling to VBSC, which in many cases, are closer to wholesale than retail or direct markets. Farmers selling to wholesale markets are often larger than the very small farm categories.

**Table 1:** Characteristics of farms in the sample

	Sample (%)		2017 Ag census (%)	
Gross farm income	(N = 255)			
Less than $1000	0		30	
$1000 to $9999	5		29	
$10,000 to $24,999	7		11	
$25,000 to $49,999	6		7	
$50,000 to $99,999	9		6	
$100,000 to $249,999	22		6	
$250,000 to $499,999	12		4	
$500,000 to $999,999	12		3	
$1,000,000 or more	27		4	
Acres operated	(N = 290)			
1 to 9 acres	19		13	
10 to 49 acres	27		29	
50 to 99 acres	14		15	
100 to 219 acres	10		17	
220 to 499 acres	11		12	
500 + acres	19		15	
Household income from farm	(N = 256)			
0%	4			
1–25%	21			
26–50%	11			
51–75%	14			
76–99%	16			
100%	34			
Commodities produced	(N = 257)			
Meats and dairy	28			
Horticultural crops	75			
Agronomic crops	24			
Regions	(N = 251)			
Pacific	35			
Northwest	23			
Northeast	22			
Southeast	6			
Midwest	14			
Gender of respondent	(N = 258)			
Female	28			
Male	72			
	(N = 257)			
	Mean	Std. dev.	Min	Max
Age of respondent (years)	53.3	13.1	23	82

In terms of the acres operated, our farmer respondents were comparable to the 2017 Ag Census distribution. The similarity in the distributions of acres farmed, combined with the divergence in the distributions of gross income, implies that our farmer respondents were grossing more per acre than the 2017 Ag Census farmers. This is consistent with the suggestion that they were likely more experienced, larger, and were more likely to farm for a significant portion of their livelihood. Indeed, 64% of our farmer survey respondents made 51% or more of their household income from the farm. It is also possible that they had higher yield per acre, produced crops with higher value per acre, or received higher price premiums from the marketing channels they used.

**Fig. 1:**
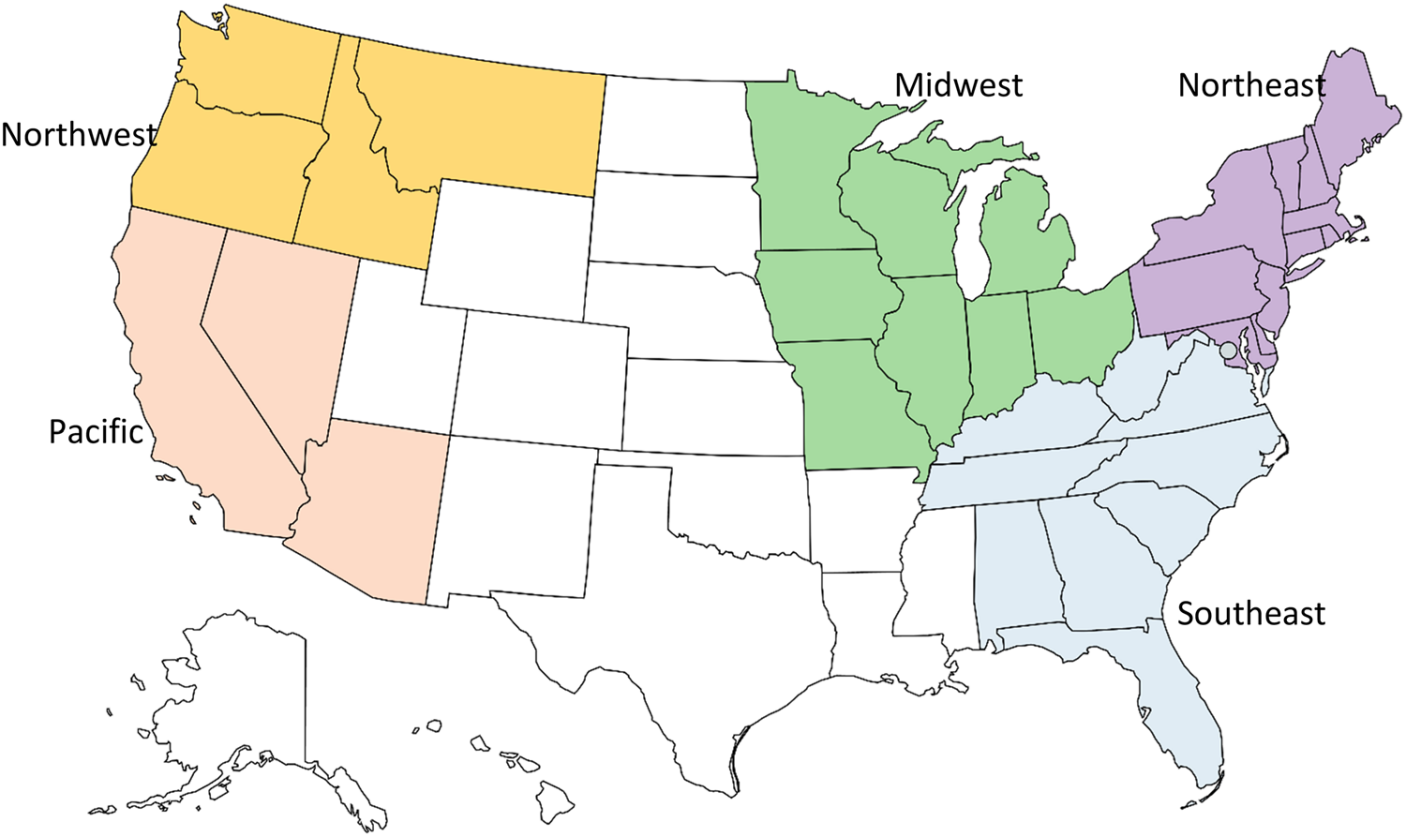
Study regions based on the USDA NASS regions

There was a good representation of types of crop and livestock products with 75% growing horticultural crops (fruits, vegetables, and nuts). Note that there were farmers producing more than one type of product. For regional distribution based on the USDA NASS regions (Fig. 1), responses from the East Mountain and Southern states were combined to create the Southeast region, and responses from the Great Lakes, Heartland, and the Upper Midwest regions were combined to create the Midwest region. Given the limited number of responses from the Mountain region, Arizona and Montana responses were added to the Pacific and Northwest regions, respectively. There were no responses received from the Plains and Delta regions. The resulting regions were well represented by our survey respondents except for the Southeast. The Pacific and Northwest had the largest representation, together accounting for 59% of the sample. The geographic distribution of our sample does not reflect the actual distribution of VBSCs in the country but rather the distribution of VBSCs that were willing to participate in the research project by sharing their lists of farmer suppliers for the survey.

The respondents tended to be male (72%), averaging 53.3 years in age with about 34 years of farming experience. Compared to the national averages for principle producers from the 2017 Agricultural Census, our respondents had a similar percentage of males (71% in the 2017 Agricultural Census) and were younger (58.6 years in the 2017 Agricultural Census) but with comparable farming experience (74% farmed 11 or more years compared to 75% in the 2017 Agricultural Census) (USDA NASS 2019).

Table 2 describes the size of our respondents’ farm operations as defined by GFI or by acres and for each of these categories, across commodities and region. Looking at GFI first, the products sold tended to be balanced across all three GFI levels. But there were relatively more farmers producing agronomic crops in the highest GFI category than those producing meats, dairy, or horticultural crops. For regional differences, there were proportionally more farmers in the Northwest grossing $500 K or more and in the Southeast grossing less than $50 K. This was because some of the Northwest VBSCs included large grain farmers, which likely increased the percentage in that category. For the Pacific, Northeast and Midwest, the largest percentages of farmers fell into the medium-scale category. Looking at the acres operated, the horticultural crop farmers (majority of our sample) tended to be farming on small acreages (up to 49 acres), suggesting relatively high values of crops. The majority of the farms in our study from the Pacific, Southeast, and Midwest regions operated fewer than 50 acres, while, due to the nature of the VBSCs surveyed, the majority of farms in the Northwest farmed 500 or more acres. The survey respondents from the Northeast were more uniformly distributed across acreage categories.

**Table 2:** Size distribution of farms by region and VBSC type

Gross farm income	Less than $50 K	$50 K or more, less than $500 K	$500 K or more	N
*Commodities produced*				
Meats and dairy	19%	47%	34%	73
Horticultural crops	16%	44%	39%	194
Agronomic crops	11%	34%	54%	61
*Region*				
Pacific	24%	39%	38%	88
Northwest	4%	28%	68%	57
Northeast	13%	63%	24%	54
Southeast	56%	38%	6%	16
Midwest	15%	55%	30%	33

Acres operated	1 to 9 acres (%)	10 to 49 acres (%)	50 to 99 acres (%)	100 to 219 acres (%)	220 to 499 acres (%)	500 + acres (%)	N
*Commodities produced*							
Meats and dairy	14	23	4	6	30	24	71
Horticultural crops	23	33	5	10	19	10	193
Agronomical crops	3	8	2	5	28	53	60
*Region*							
Pacific	28	35	18	4	11	3	89
Northwest	0	15	11	13	9	53	55
Northeast	11%	30	11	17	15	15	53
Southeast	31	38	13	13	6	0	16
Midwest	21	33	6	12	12	15	33

### How important are VBSCs for the participating farms?

Table 3 summarizes our three outcome variables on how important the VBSC is to the farm, tabulated by GFI and by acres operated. According to the top panel of the table, the smallest farms by GFI tended to sell the highest percentage of their sales to the VBSC, averaging 43%, while the largest farms on average sold 16% of their crop sales through the VBSC. The range of percentage of sales to the VBSC was wide across all three GFI categories. Two out of five of the smallest farms ranked the VBSC as their most preferred outlet, compared to barely one in four among mid-size and large farms. Regarding their marketing portfolio, more than two-thirds of the small and mid-size farms included their VBSC within their top three most preferred marketing channels, compared to 42% of the largest farms.

**Table 3:** Use of VBSC by farm size

By gross farm income	Less than $50 K	$50 K or more, less than $500 K	$500 K or more
Average percentage of sales sold to VBSC	43	25	16
Ranks VBSC as the most preferred	40%	25%	23%
Ranks VBSC among the top 3 most preferred	67%	70%	42%

By acres operated	1 to 9 acres	10 to 49 acres	50 to 99 acres	100 to 219 acres	220 to 499 acres	500+ acres
Average percentage of sales sold to VBSC	32	26	22	25	16	17
Ranks VBSC as the most preferred	20%	23%	24%	28%	28%	35%
Ranks VBSC among the top 3 most preferred	63%	57%	47%	52%	47%	60%

When we look at the same data in the bottom panel, the values tend to be more evenly distributed across acreage categories. The farms under 10 acres sold on average 32% of their crops to the VBSC, compared to the farms with 500 acres or more which sold on average 17%. The percentages of farms ranking their VBSC as the most important outlet ranged from 20 to 35%, with the largest percentage among the very largest farms (by acreage). Since we know these farms sell mostly agronomic crops, we suggest that these farmers found the VBSC to be particularly useful and valued. In general, the majority of farms across the size categories by acreage included their VBSC among the top three most important channels, with the percentages of farms ranging from 47 to 63%.

In Table 4, we take a detailed look at how farms rank the importance of various marketing outlets to gain further insights into how farmers are using their VBSC in their marketing portfolio. The data are again organized by GFI first, and by acres operated second. The number of respondents including the outlet within their top three choices are reported for the entire sample and by size categories. The ranking is reported based on the scores where values 1, 2, and 3 are assigned to the top, second, and third choices, respectively. VBSC was included among the top three choices by the largest number of farmers (N = 179 or 61%) in our sample, followed by direct sales (N = 167) and retail outlets (N = 160). When looking at the tabulation by GFI, we see that the smallest and medium sized farms ranked their VBSC as the most important outlet for their products, while the largest farms ranked conventional wholesale buyers as the most important and VBSCs are ranked second. When organized by acres operated, smaller and medium scale farms up to 100 acres rank VBSCs as their most important marketing outlet. Its ranking fluctuated among bigger farms but was maintained among the top three.

**Table 4:** Ranking of importance of marketing outlets by farm size

By gross farm income	All	Less than $50 K			$50 K or more, less than $500 K			$500 K or more		
	N^*a*^	Ranking^*b*^	Average score^*c*^	N	Ranking	Average score	N	Ranking	Average score	N
Values-based supply chains	179	1	1.57	35	1	1.76	74	2	1.77	70
Direct sales to individual consumers^*d*^	167	4	2.04	26	3	1.85	81	3	1.83	60
Retail outlets^*e*^	160	6	2.33	24	6	2.17	65	5	2.14	71
Wholesale buyers, brokers, or packers	141	3	1.75	16	2	1.82	39	1	1.74	86
Food cooperatives	49	2	1.67	9	4	2.05	20	6	2.45	20
Growers/farmers cooperatives	42	5	2.25	4	5	2.17	12	4	2.08	26

By acres operated	1 to 9 acres			10 to 49 acres			50 to 99 acres			100 to 219 acres			220 to 499 acres			500 + acres		
	Ranking^*b*^	Average score^*c*^	N^*d*^	Ranking	Average score	N	Ranking	Average score	N	Ranking	Average score	N	Ranking	Average score	N	Ranking	Average score	N
Values-based supply chains	1	1.75	40	1	1.66	47	1	1.63	19	3	2.00	18	1	1.64	11	2	1.73	44
Direct sales to individual consumers^*d*^	4	1.89	38	2	1.74	58	3	1.75	20	2	1.88	16	2	1.88	17	6	2.39	18
Retail outlets^*e*^	6	2.14	36	5	2.27	49	4	2.32	19	5	2.08	12	4	1.95	21	5	2.22	23
Wholesale buyers, brokers, or packers	3	1.87	15	3	1.77	26	2	1.75	24	1	1.78	18	3	1.90	20	1	1.66	38
Food cooperatives	2	1.86	14	4	2.23	13	5	2.43	7	4	2.00	5	6	2.33	9	4	2.00	1
Growers/farmers cooperatives	5	2.00	1	6	2.50	4	6	2.60	5	6	2.50	6	5	2.33	3	3	1.83	23

^*a*^Number of respondents including the outlet within their top three choices

^*b*^Ranking based on the average score

^*c*^1 = top choice, 2 = second choice, 3 = third choice

^*d*^Including roadside stands, farm stores or U-pick sales, farmers markets, Community Support Agriculture, mail order, or Internet

^*e*^Including restaurants, grocery stores, schools, hospitals, or other businesses) that in turn sell directly to consumers

Conventional wholesale was ranked third among the smallest farms, second among the mid-sized farms, and the first among the largest farms by GFI. A similar trend was observed based on acreage with the exception of the 220 to 499 acre category. Direct sales to consumers are ranked third among larger farms with the average score of 1.83, suggesting that there are large farms that regard this channel as their top priority. Direct sales to consumers was ranked fourth among the smallest farms with the average score of 2.04, suggesting that proportionally fewer farms in the smallest category compared to the largest farm category prioritized direct sales to consumers. In terms of acreage, direct sales is important for farms between 10 and 500 acres, but not as important among the smallest and largest farms. The other marketing outlets were ranked below VBSCs, conventional wholesale, and direct sales, by farms across size with the exception of food cooperatives favored by the smallest farms grossing less than $50 K or with fewer than 10 acres.

### What are the benefits and challenges in participating in the VBSC(s)?

Table 5 summarizes the farmers’ binary responses to benefits and challenges of selling to VBSCs. The average number of responses differ notably between benefits and challenges, because respondents were first asked to identify whether they benefit or face any challenges from marketing through their VBSC and only those that said “yes” proceeded to consider individual items. Benefits are categorized by organizational, promotional, and values-based benefits. The highest percentage (88%) among those who perceived benefits were in agreement with the statement that the VBSC fits with my values (values-based benefit), followed by 79% of farmers who said VBSCs were predictable and provided timely payments (organizational benefit) and then 81% who said VBSCs provided access to new or larger markets (promotional benefit).

**Table 5:** Benefits from and challenges of selling through VBSC

	%Respondents perceiving benefits/challenges	N
*Benefits from marketing through VBSC*		
Organizational benefits		
1. Receive a premium for my products	53	227
2. Technical assistance regarding farming practices from VBSC	13	224
3. Marketing services from VBSC	58	226
4. Predictable and/or timely payments	79	227
Promotional benefits		
5. Access to new or larger markets	81	227
6. Network with other farmers	35	225
7. Strengthened connections with other businesses in the supply chain	47	226
8. Strengthened identity in the marketplace	72	225
Values-based benefits		
9. Fits with my values	88	222
10. My environmental values are communicated to consumers	65	217
11. My commitment to the well-being of my community is communicated to consumers	64	213
*Challenges*		
Organizational challenges		
1. They won't take enough volume	69	132
2. Transportation and delivery logistics	36	134
3. Variable and/or delayed payments	24	134
Required standards		
4. Required production practices	17	134
5. Quality standards	22	132
6. Labor standards	7	134
7. Organic certification	8	130
8. Food safety regulations	19	134
9. Animal welfare standards	2	116
Operational challenges		
10. I don't have enough volume	26	131
11. Finding enough, qualified labor	22	134

The number of farmers indicating they faced challenges in working with VBSCs were just about half of the number of farmers identifying benefits. The largest percentage (69%) of those who identified challenges said VBSCs would not take enough of their product. The next highest complaint was that transportation and delivery logistics with the VBSC were difficult (36%).

The benefits were aggregated into three benefit factors using the scoring coefficients based on factor loading reported in Table 10 in Appendix. The organizational challenges, acknowledged by the largest percentages of responses, were not correlated enough to be represented by a single factor. Given the low variability in responses regarding challenges, we decided to look at a few selected challenges independently and a factor for required standards based on three items (required production practices, quality standards, and labor standards, i.e., challenge items 4, 5, and 6 in Table 5). Selected challenges include VBSC not taking enough volume, transportation and delivery logistics, and farmers not having enough volume (challenge items 1, 2, and 10 in Table 5).

### Regression analysis

The results from regression analyses are reported in terms of average marginal effects in Tables 6, 8, and 9. To examine the impact of farm size on the use, benefits, and challenges associated with VBSCs in comparable terms, elasticities with respect to the three farm structure variables are summarized in Table 7. The regression coefficients are reported in the Appendix Tables 11, 12, and 13.

**Table 6:** Average marginal effects on use and importance variables

	*pct_sold*	*vbsc_rank1*	*vbsc_top3*
Farm characteristic			
*acres*	− 0.238**	− 0.010	0.045*
	(0.107)	(0.009)	(0.025)
*gfi*	− 0.059***	0.000	− 0.002***
	(0.022)	(0.001)	(0.001)
*pct_farminc*	− 0.030	0.000	0.001
	(0.037)	(0.001)	(0.002)
Commodities produced			
*meats&dairy*	3.535	− 0.053	− 0.062
	(3.250)	(0.055)	(0.066)
*hortcrop*	− 0.148	− 0.189***	− 0.096
	(4.466)	(0.058)	(0.079)
*agroncrop*	− 9.882***	0.149	0.039
	(3.105)	(0.111)	(0.072)
Region (base = *Midwest*)			
*Northwest*	3.640	− 0.123	− 0.037
	(3.723)	(0.099)	(0.132)
*Pacific*	− 4.368	− 0.088	− 0.132
	(4.337)	(0.128)	(0.124)
*Northeast*	1.503	− 0.195*	− 0.230
	(3.747)	(0.117)	(0.145)
*Southeast*	13.831**	0.092	− 0.209*
	(5.686)	(0.114)	(0.121)
Operator characteristic			
*female*	− 6.627**	− 0.044	− 0.128**
	(2.929)	(0.045)	(0.056)
*age*	− 0.117	0.004*	0.002
	(0.102)	(0.003)	(0.002)
Number of obs	225	226	226
p-value for F/χ^2^ test	0.000	0.000	0.000
Pseudo R-squared	0.014	0.086	0.101

*, **, and *** signify statistical significance at the 0.1, 0.05, and 0.01 levels, respectively

The numbers reported for the *pct_sold* equation are average marginal effects based on a tobit regression, accounting for the probability of the dependent variable censored at 0 and 100

The numbers reported for the *vbsc_rank1* and *vbsc_top3* equations are average marginal effects based on a logistic regression. The numbers in parentheses are standard errors adjusted for VBSC clusters

**Table 7:** Average elasticities with respect to farm size

	*acres*	*gfi*
Use and importance		
*pct_sold*	− 0.008	− 0.131**
	(0.007)	(0.054)
*vbsc_rank1*	− 0.027	0.025
	(0.034)	(0.171)
*vbsc_top3*	0.027	− 0.278**
	(0.018)	(0.120)
Benefit factors		
*Organizational*	0.023***	− 0.019
	(0.008)	(0.030)
*Promotional*	0.011**	− 0.020
	(0.004)	(0.051)
*Values-based*	0.057***	− 0.025
	(0.017)	(0.075)
Challenges		
*Standards*	0.033	− 0.054
	(0.020)	(0.055)
*VBSC limits volume*	0.024	− 0.040
	(0.026)	(0.061)
*Logistics*	0.059	− 0.129
	(0.041)	(0.198)
*Farm volume*	− 0.044	− 0.849***
	(0.095)	(0.266)

*, **, and *** signify statistical significance at the 0.1, 0.05, and 0.01 levels, respectively

The numbers in parentheses are standard errors adjusted for VBSC clusters

The results for the three outcome variables measuring the use and importance of VBSCs by farmers are reported in Table 6. All else equal, smaller farms in terms of both acreage and GFI generate greater percentages of their sales through the VBSC. The estimated magnitudes, albeit statistically significant, suggest small impacts, where the average change is 0.24 percentage points for a difference of 1,000 acres (p = 0.026) and 0.06 percentage points for a difference of $10,000 in GFI (p = 0.007). Elasticity with respect to acres is not statistically significant, but a 1% decrease in GFI is associated with a 0.13 percent increase in proportion of crops sold through the VBSC (Table 7, p = 0.016). The recognition of the VBSC as the most important marketing channel did not vary across farm structure, but farms grossing less and farms with greater acreage were both more likely to include the VBSC in their top three important marketing channels, all else equal (p = 0.001 and 0.066, respectively).

Examining farms by the type of product sold, producers of agronomic crops sold nearly 10 percentage points less of their crops through the VBSC than those who do not produce agronomic crops, all else equal (p = 0.001). Horticultural crop producers were less likely to rank VBSC as their top marketing channel than their counterparts. There were no statistically significant differences across regions, except for producers in the Southeast selling 13.8 percentage point more of their crops (p = 0.015) and less likely to include their VBSC among their top three most important marketing channels (p = 0.083). There were also slight tendencies among farmers in the Northeast to not consider their VBSC as their most important marketing channel (p = 0.096).

Table 8 summarizes the results for the differences across farms on perceived benefit factors. Regarding farm structural characteristics, holding everything else equal, farms with more acres were more likely to express perceived benefits of all kinds considered in this study, but in particular, values-based and organizational benefits, suggesting a 0.18 and 0.06% increase respectively in these perceived benefits for each additional 1,000 acres (p = 0.002 and  < 0.001) or 0.06 percent and 0.02 percent increase respectively for a percent increase in acreage (Table 7, p = 0.001 and 0.002). There were no statistical differences across farms grossing different amounts or relying differently on other income sources. The additional statistical differences were found only for organizational benefits. Farmers who were less likely to express perceived organizational benefits included horticultural crop producers (p = 0.032), producers in the Northeast relative to those in the Midwest (p = 0.042), and female operators (p = 0.006).

**Table 8:** Average marginal effects on benefit factors

	Organizational	Promotional	Values-based
Farm characteristic			
*acres*	0.061***	0.014*	0.175***
	(0.014)	(0.007)	(0.057)
*gfi*	− 0.0003	− 0.0004	− 0.0005
	(0.0005)	(0.0009)	(0.0013)
*pct_farminc*	0.000	0.001	0.003
	(0.001)	(0.001)	(0.004)
Commodities produced			
*meats&dairy*	0.033	0.127	− 0.113
	(0.055)	(0.094)	(0.148)
*hortcrop*	− 0.190**	− 0.138	− 0.194
	(0.081)	(0.124)	(0.157)
*agroncrop*	0.038	0.179	0.131
	(0.108)	(0.128)	(0.294)
Region (base = *Midwest*)			
*Northwest*	− 0.066	0.185	− 0.011
	(0.133)	(0.220)	(0.350)
*Pacific*	− 0.030	0.110	0.204
	(0.067)	(0.180)	(0.287)
*Northeast*	− 0.157**	0.057	− 0.094
	(0.071)	(0.192)	(0.221)
*Southeast*	0.059	0.075	0.247
	0.067	(0.162)	(0.264)
Operator characteristic			
*female*	− 0.124***	0.017	0.106
	(0.039)	(0.074)	(0.113)
*age*	0.000	− 0.004	0.005
	(0.002)	(0.003)	(0.007)
Number of obs	204	200	200
p-value for F test	0.000	0.000	0.000
R-squared	0.190	0.101	0.083

*, **, and *** signify statistical significance at the 0.1, 0.05, and 0.01 levels, respectively

The numbers in parentheses are standard errors adjusted for VBSC clusters

The dependent variables are natural logarithms of factors generated from factor analysis, translated by 1.5

Table 9 summarizes the results for challenges. All else equal, farmers with greater acreage were more likely to identify required standards to market through their VBSC as a challenge (p < 0.001), while horticultural crop producers were less likely to identify it as such (p = 0.039). Agronomic crop producers and farmers in the Pacific regions were more likely to indicate that their VBSC limited the purchase volume, although farmers were interested in selling more (p = 0.008 and < 0.001, respectively). Logistics was identified as a challenge by the second largest proportion of respondents, but no statistical differences were found across different farm characteristics, except for a slight association with the operator’s age. Farms grossing more and those producing meats and dairy products were less likely to identify not having enough volume to sell through their VBSC (p = 0.001 and < 0.001, respectively). One percent increase in GFI was associated with 0.85 percent decrease in the likelihood of the farmer saying that not producing enough volume was a challenge (p = 0.001, Table 7).

**Table 9:** Average marginal effects on challenge factors/items

	Standards	VBSC limits volume	Logistics	Farm volume
Farm characteristic				
*acres*	0.035***	0.041	0.047	− 0.007
	(0.006)	(0.050)	(0.042)	(0.014)
*gfi*	− 0.001	− 0.001	− 0.004	− 0.003***
	(0.001)	(0.001)	(0.006)	(0.001)
*pct_farminc*	0.001	0.002	0.000	0.000
	(0.001)	(0.001)	(0.001)	(0.001)
Commodities produced				
*meats&dairy*	− 0.044	0.026	0.014	− 0.252***
	(0.072)	(0.070)	(0.144)	(0.050)
*hortcrop*	− 0.334 **	0.058	− 0.119	0.112
	(0.150)	(0.109)	(0.131)	(0.103)
*agroncrop*	− 0.048	0.207***	− 0.014	− 0.063
	(0.105)	(0.078)	(0.133)	(0.075)
Region (base = *Midwest*)				
*Northwest*	0.060	0.036	0.036	− 0.049
	(0.140)	(0.111)	(0.114)	(0.111)
*Pacific*	− 0.109	0.356***	− 0.087	0.056
	(0.152)	(0.099)	(0.130)	(0.089)
*Northeast*	0.139	− 0.004	− 0.165	0.043
	(0.236)	(0.075)	(0.199)	(0.076)
*Southeast*	0.313	− 0.244	0.238	0.146
	(0.255)	(0.234)	(0.224)	(0.094)
Operator characteristic				
*female*	0.117	0.003	0.122	− 0.224
	(0.068)	(0.117)	(0.094)	(0.137)
*age*	0.005	− 0.001	0.004*	0.002
	(0.004)	(0.003)	(0.002)	(0.003)
Number of obs	120	119	119	119
p-value for F/χ^2^ test	0.000	0.000	0.000	0.000
R-squared/Pseudo R^2^	0.243	0.183	0.106	0.234

*, **, and *** signify statistical significance at the 0.1, 0.05, and 0.01 levels, respectively

The numbers in parentheses are standard errors adjusted for VBSC clusters

The *fstd3* equation is estimated with OLS, where the dependent variable is a natural logathrism of the factor translated by 1. The other three equations are estimated with logit regression

## Discussion

Our survey confirms that the AOTM farms in our study are pursuing unique strategies with respect to how they incorporate VBSCs into their marketing portfolios. Furthermore, farms that use VBSCs encompass more than conventionally identified AOTM farms, which perhaps is indicative of the evasive notion of the “middle” sector of agriculture as discussed below. When we examine all these results together, three key insights emerge concerning: (a) the use and importance of the participating VBSC to farmers’ businesses, (b) benefits and challenges of selling through VBSCs from farmers’ perspectives, and (c) the variability of farmers’ experiences.

First, our findings confirm that VBSCs are valued by the majority of farmers who include them in their marketing portfolios and consider them relatively more important compared to other marketing outlets. Second, nearly all (90%) respondents indicated that they benefited from selling through their VBSC, while half (51%) of respondents indicated they face some challenges in selling through their VBSC. Farmers find VBSCs valuable because of their relational and cultural values beyond simple economic gains or business conveniences. Along with organizational benefits, which include marketing services provided by the VBSC, predictable and/or timely payments, and access to new or larger markets; the farmers appreciated the promotional benefit of having a stronger identity in the marketplace. More than a third of respondents perceived benefits from networking with other farmers and nearly half valued strengthened connections with other businesses in the supply chain. Values-based benefits such as a sense of shared values with the business partners and having the farmers’ values communicated to consumers through the VBSC were perceived by the highest proportion of producers. A higher percentage agreed to the general “fits with my values” than specific values associated with the environment or community, suggesting that perceived values-based benefits are more nuanced. These relational values differentiate VBSCs from conventional marketing channels, and the findings show that the farmers appreciate these aspects of VBSCs.

Third, the variability in responses rejects a “one-size-fits all” scenario and calls for a more refined examination. Although we will describe statistically significant trends below, the magnitude of such trends is not always strong. The picture of how farmers interact with their VBSC is much more modulated than we had anticipated. We will address the variability across farms of various size, product type, and regions in turn.

### Variability in experience across farm size

Our analysis validates the claim that farm size matters in terms of how farmers incorporate VBSCs into their marketing portfolios, how important the VBSC is to the operation, and the benefits and challenges faced. The regression analyses confirm the trends suggested by descriptive results where smaller farms (both in terms of GFI and acres) are more likely to sell a higher percentage of overall sales to their VBSC. The smaller farms are also more likely to rank their VBSC as one of their top three marketing channels. Yet, it is the larger farms (in terms of acres operated) that tend to perceive more of the three types of benefits identified—organizational, promotional and values-based. It may be that smaller farms are already connected with other direct markets that provide these benefits, whereas larger farms have identified fewer direct market options and may be relying on the VBSC to provide these same benefits. Lastly, larger farms are more likely to report that standards such as quality and labor standards and organic certification are challenges when selling to their VBSCs, while smaller farms are more likely to report that they do not have enough volume when selling to their VBSC.

Elasticities reported in Table 7 allow us to look at the impact of farm size in comparable terms. What the table reveals is that when controlling for types of products produced or regions, the use and importance varies across farms of different size, measured by GFI but not by acreage. In contrast, farm size in acres (not GFI) is positively associated with all benefit factors considered in the study. We suggest that larger farms may not have other marketing channels that provide these benefits as compared to smaller farms. Smaller farms (by GFI, not acres), however, are more likely to be challenged by not having enough volume.

### Variability in experience across product types and regions

The use and importance of VBSCs and associated benefits and challenges varied across product categories sold by farmers and regions as well. Agronomic crops (grains, beans) are much less likely to be sold through VBSCs and horticultural crops (fruits, vegetables, nuts) are less likely to rank the VBSC as their top marketing outlet. We suggest that both types of crops may have comparatively better marketing outlets (perhaps larger wholesale markets for agronomic crops and more direct markets for horticultural crops). Farmers from the Southeast were more likely to sell their products through VBSCs. Northeast farmers were slightly less likely to rank their VBSC as the most important channel. Further, farms selling horticultural crops and Northeastern farmers were less likely to realize organizational benefits of VBSCs, consistent with our hypothesis that could be explained by the presence of other marketing channels available to these farmers. We surmise these regional variations may come from unique regional histories in which the VBSCs included in our study were formed and built relationships with farmers.

It is interesting to note that limits to volume (e.g., the VBSC not taking enough volume) is more of a challenge for agronomic crops (grains, beans) and especially for the scale of the Pacific farmers in our study. We assume that farms selling crops like grains and beans might be used to selling very large quantities in commodity markets and perhaps smaller VBSCs may not be able to accommodate those same volumes. For farmers in the Pacific region, there may be higher demands for selling through VBSCs and if VBSCs were saturated, they might not be able to accommodate the volume. Meat and dairy farmers, on the other hand, are less likely to be challenged by having sufficient volume to sell to the VBSCs. Farms selling horticultural crops are less likely to be challenged by these standards specific to selling through their VBSC. They have been the subject of much education and training over the last five years as the USDA prepared the rule on standards for produce safety required by the Food Safety Modernization Act (FSMA). It is also possible that farms that are opting-in to VBSCs with certifications may already have chosen those certifications or practices independent of their choice to sell through VBSC (Brekken et al. 2017).

This study is not representative of the full range of VBSCs in the United States. While we tried our best to identify and build a comprehensive list of all the VBSCs in the U.S., because this is an evolving concept, there was no pre-existing list to compare our list against. Also, because the farmer lists are proprietary, the full cooperation of the VBSC leaders was required in order to survey their farmer members. Filling out surveys is not very popular with farmers, a factor that may have made some VBSC leaders reluctant to participate or to promote the study with their farmer members. Given these challenges we are grateful to the VBSC leaders who did agree to partner with us and the relatively high number of farmers who filled out the survey.

## Conclusion

Our survey reveals the complexity of farmers’ motivations for participating in VBSCs as well as their perceptions of the benefits and challenges of VBSCs. Because VBSCs provide aggregation services, there is a tendency to assume that their intended farm clients are small and medium-sized. Our findings confirm that VBSCs are not only serving small and medium-sized farms but also large farms. For smaller farms, it is an important outlet accounting for a higher percentages of their sales. But it is the larger farms that are more likely to perceive VBSC-specific benefits.

On the one hand, our study showed that the importance the VBSCs play for farmers differed by operation size, types of crops grown, or regions. Underlying reasons for these differences may be due to the availability of other differentiated marketing options for individual farmers and the flexibility of the farm’s production. Although we began our investigation to understand the contributions of VBSCs to serving the middle-sector, our findings about VBSCs’ farmers suggest the need for reevaluating the “middle” through additional research that incorporates farmer perspectives. Future research could include qualitative approaches that focus on understanding farmer perceptions about their marketing strategies and the performance of various markets in relation to farm size and type.

On the other hand, the evasiveness of the “middle” as an empirical category in the structure of US agriculture suggests that the “middle” may require additional criteria beyond GFCI and acreage, particularly when conceptualizing the AOTM as something between the LFSs/SFSCs and global commodity systems/long food supply chains. Regional comparisons of farms with similar cropping or livestock systems through qualitative methods could be useful in refining the framing of the “middle” as a conceptual and empirical category. Moreover, future research needs to examine regional specificities in how VBSCs emerge and operate as business entities, how they build their relational space with farmers, and the effects of various regulatory and policy environments on farm scale, conservation strategies, and marketing choices. Findings from such an investigation will contribute to designing more targeted policy changes as well as extension and educational programming that support the development of regional supply chains.

The usefulness of regional supply chains is inherently limited by the overall regional market. Our findings substantiate what we have heard anecdotally that there is limited volume of product that regional supply chains can handle. Conceptually, for larger scale farms with high-volume crops, there seems to be an inherent conflict between the volumes that farmers need to market and what the VBSCs can sell regionally. Despite the volume limitation, our study confirms that VBSC is a valuable option as part of a mixture of diverse strategies found in farmer marketing portfolios.

The importance of VBSCs and regional supply chains is even more heightened now given the enormous shock to the food system from the COVID-19 pandemic and its impact on farmers. Various forms of intermediating businesses that can connect agricultural producers to consumers, while preserving the distinctive identities of the products and facilitating fair business relationships along the supply chain, appear to offer valuable market alternatives for small and medium-sized farmers, as well as some larger farmers.

### Electronic supplementary material

Below is the link to the electronic supplementary material.

Supplementary file1 (DOCX 32 kb)

## Data Availability

The survey instrument is available from authors upon request.

## Appendix

See Tables 10, 11, 12, and 13.

**Table 10:** Scoring coefficient used to predict benefit and challenge factors

Organizational		Promotional	
B1. premium	0.2639	B5. access	0.1427
B2. techassist	0.3194	B6. connection	0.4032
B3. service	0.2631	B7. identity	0.1471
B4. payments	− 0.0428	B8. network	0.3209

Values-based		Standards	
B9. values	0.1036	C4. practices	0.2948
B10. envvalues	0.4651	C5. quality	0.3007
B11. community	0.4319	C6. laborstds	0.3822

The item numbers correspond to Benefits and Challenges listed in Table 5

The coefficients are based on varimax rotated factors

**Table 11:** Regression results for use and importance variables

	*pct_sold*	*vbsc_rank1*	*vbsc_top3*
Intercept	42.812***	− 1.009	1.636*
	(8.657)	(1.065)	(0.994)
Farm characteristic			
*acres*	− 0.299**	− 0.056	0.228
	(0.136)	(0.048)	(0.140)
*gfi*	− 0.074***	0.001	− 0.012***
	(0.026)	(0.004)	(0.004)
*pct_farminc*	− 0.038	0.000	0.005
	(0.047)	(0.005)	(0.008)
Commodities produced			
*meats&dairy*	4.447	− 0.286	− 0.309
	(4.140)	(0.297)	(0.339)
*hortcrop*	− 0.187	− 1.016***	− 0.480
	(5.620)	(0.276)	(0.397)
*agroncrop*	− 12.434***	0.802	0.194
	(3.921)	(0.616)	(0.359)
Region (base = *Midwest*)			
*Northwest*	4.580	− 0.658	− 0.185
	(4.667)	(0.534)	(0.663)
*Pacific*	− 5.495	− 0.470	− 0.662
	(5.448)	(0.700)	(0.630)
*Northeast*	1.891	− 1.046*	− 1.155
	(4.706)	(0.605)	(0.724)
*Southeast*	17.402 **	0.496	− 1.049*
	(7.255)	(0.620)	(0.620)
Operator characteristic			
*female*	− 8.337**	− 0.237	− 0.643**
	(3.748)	(0.242)	(0.312)
*age*	− 0.147	0.024*	0.009
	(0.128)	(0.014)	(0.012)
Number of obs	225	226	226
p-value for F/χ^2^ test	0.000	0.000	0.000
Pseudo R-squared	0.014	0.086	0.101

*, **, and *** signify statistical significance at the 0.1, 0.05, and 0.01 levels, respectively

The numbers in parentheses are standard errors adjusted for VBSC clusters. The *pct_sold* equation is estimated using tobit regression with the dependent variable censored at 0 and 100. The *vbsc_rank1* and *vbsc_top3* equations are estimated using logit regression

**Table 12:** Regression results for benefit factors

	Organizational	Promotional	Values-based
Intercept	0.505	0.400	− 0.461
	(0.127)	(0.250)	(0.517)
Farm characteristic			
*acres*	0.081***	0.014*	0.181***
	(0.023)	(0.007)	(0.056)
*acres*^2^	− 0.001***		− 0.004***
	(0.000)		(0.001)
*gfi*	− 0.0003	− 0.0004	− 0.0005
	(0.0005)	(0.0009)	(0.0013)
*pct_farminc*	0.000	0.001	0.003
	(0.001)	(0.001)	(0.004)
Commodities produced			
*meats&dairy*	0.033	0.127	− 0.113
	(0.055)	(0.094)	(0.148)
*hortcrop*	− 0.190**	− 0.138	− 0.194
	(0.081)	(0.124)	(0.157)
*agroncrop*	0.038	0.179	0.131
	(0.108)	(0.128)	(0.294)
Region (base = *Midwest*)			
*Northwest*	− 0.066	0.185	− 0.011
	(0.133)	(0.220)	(0.350)
*Pacific*	− 0.030	0.110	0.204
	(0.067)	(0.180)	(0.287)
*Northeast*	− 0.157**	0.057	− 0.094
	(0.071)	(0.192)	(0.221)
*Southeast*	0.059	0.075	0.247
	(0.067)	(0.162)	(0.264)
Operator characteristic			
*female*	− 0.124***	0.017	0.106
	(0.039)	(0.074)	(0.113)
*age*	0.000	− 0.004	0.005
	(0.002)	(0.003)	(0.007)
Number of obs	204	200	200
p-value for overall F test	0.000	0.000	0.000
R-squared	0.190	0.101	0.083

*, **, and *** signify statistical significance at the 0.1, 0.05, and 0.01 levels, respectively

The numbers in parentheses are standard errors adjusted for VBSC clusters. The dependent variables are natural logarithms of factors generated from factor analysis, translated by 1.5

**Table 13:** Regression results for challenge factors/items

	Standards (factor)	VBSC limits volume	Logistics	Farm volume
Intercept	− 0.310	− 0.507	− 1.204	− 0.885
	(0.265)	(1.522)	(1.135)	(1.919)
Farm characteristic				
*acres*	0.035***	0.238	0.231	− 0.049
	(0.006)	(0.304)	(0.200)	(0.101)
*gfi*	− 0.001	− 0.003	− 0.004	− 0.019
	(0.001)	(0.004)	(0.006)	(0.005)
*pct_farminc*	0.001	0.010	0.000	0.001
	(0.001)	(0.006)	(0.007)	(0.006)
Commodities produced				
*meats&dairy*	− 0.044	0.151	0.068	− 1.792
	(0.072)	(0.416)	(0.710)	(0.372)
*hortcrop*	− 0.334**	0.342	− 0.588	0.799
	(0.150)	(0.647)	(0.662)	(0.718)
*agroncrop*	− 0.048	1.210***	− 0.068	− 0.445
	(0.105)	(0.471)	(0.658)	(0.531)
Region (base = *Midwest*)				
*Northwest*	0.060	0.210	0.180	− 0.347
	(0.140)	(0.651)	(0.572)	(0.789)
*Pacific*	− 0.109	2.086***	− 0.431	0.396
	(0.152)	(0.546)	(0.635)	(0.645)
*Northeast*	0.139	− 0.024	− 0.819	0.309
	(0.236)	(0.441)	(1.002)	(0.549)
*Southeast*	0.313	− 1.429	1.179	1.036
	(0.255)	(1.412)	(1.136)	(0.690)
Operator characteristic				
*female*	0.117	0.019	0.606	− 1.595
	(0.068)	(0.685)	(0.448)	(1.051)
*age*	0.005	− 0.006	0.020 *	0.012
	(0.004)	(0.017)	(0.012)	(0.022)
Number of obs	120	119	119	119
p-value for F/χ^2^ test	0.000	0.000	0.000	0.000
R-squared/Pseudo R^2^	0.236	0.183	0.106	0.234

*, **, and *** Signify statistical significance at the 0.1, 0.05, and 0.01 levels, respectively

The numbers in parentheses are standard errors adjusted for VBSC clusters

The *standard* equation is estimated with OLS. The other three equations are estimated with logit regression

## Abbreviations

VBSC: Values-based supply chains
AOTM: Agriculture of the middle
GFI: Gross farm income
GCFI: Gross cash farm income
LFS: Local food system
SFSC: Short food supply chains

## Code availability

The Stata code used for analysis is available from authors upon request.
