# Supplementary material for: The value of values-based supply chains: farmer perspective
Source: Agric Human Values. 2021 Aug 23;39(1):385–403. doi: 10.1007/s10460-021-10255-5 (PMC8382098; doi:10.1007/s10460-021-10255-5)
Supplement: Supplementary file 1 — Supplementary file1 (DOCX 32 kb) [file 10460_2021_10255_MOESM1_ESM.docx]

OMB No. 0925-0001 and 0925-0002 (Rev. 09/17 Approved Through 03/31/2020)

BIOGRAPHICAL SKETCH

Provide the following information for the Senior/key personnel and other significant contributors.
Follow this format for each person. **DO NOT EXCEED FIVE PAGES.**

NAME: Feenstra, Gail Whiting

eRA COMMONS USER NAME (credential, e.g., agency login):

POSITION TITLE: Director, University of California Sustainable Agriculture Research and Education Program (SAREP), Agriculture and Natural Resources (ANR)

EDUCATION/TRAINING (Begin with baccalaureate or other initial professional education, such as nursing, include postdoctoral training and residency training if applicable. Add/delete rows as necessary.)

| INSTITUTION AND LOCATION | DEGREE  (if applicable) | Completion Date  MM/YYYY | FIELD OF STUDY |
| --- | --- | --- | --- |
| Columbia University Teacher’s College | M.S. | 05/1983 |  |
| Columbia University Teacher’s College | M.Ed. | 05/1984 |  |
| Columbia University Teacher’s College | Ed.D. | 08/1986 |  |

**A. Personal Statement**

**B. Positions and Honors**

**Professional Experience**

**1989-2020 Deputy Director, Food Systems Coordinator, University of California Sustainable Agriculture Research and Education Program (SAREP)/ Agricultural Sustainability Institute (ASI), University of California, Davis**

**2019-2020 Acting Director, Agricultural Sustainability Institute, UC Davis**

**2020 - Director, University of California Sustainable Agriculture Research and Education Program**

**Professional Activities**

**2014- Co-chair, California Communities/Food Systems Program Team, UCANR**

**2016-2017 Guest editor, California Agriculture, Special Issue (Global Food Initiative)**

**2017-2018 Editorial Board, Special Issue of Health Promotion Practice Journal**

**2018-2021 Board member, Treasure, Agriculture, Food and Human Values Society**

**Professional Memberships**

**Registered dietitian, Academy of Nutrition and Dietetics**

**Agriculture, Food and Human Values Society**

**Society for Nutrition Education and Behavior**

**C. Contributions to Science**

**Publications (selected, last 2 years)**

**1. Feenstra, G. (2019). Increasing the capacity for place-based food systems. (Place- based food systems keynote address, Place-based Food Systems Conference, Kwantlen Polytechnic University, Vancouver BC, Canada.) Journal of Agriculture, Food Systems, and Community Development, Advance online publication.**

**https://doi.org/10.5304/jafscd.2019.091.018.**

**2. Brekken, C., Dickson, C., Peterson, H., Feenstra, G., Tanaka, K., Ostrom, M., and Engelskirchen, G. (2019). Economic impact of values-based supply chain participation on small and mid-sized produce farms. Journal of Food Distribution Research, Volume 50, Issue 2, pp1-26. . ISSN 2643-3354 (online); https://www.fdrsinc.org/wp-content/uploads/2019/11/JFDR_50.2.pdf#page=6**

**3. Gupta, C., Campbell, D., Munden-Dixon, K., Sowerwine, J., Capps, S., Feenstra, G., and Van Soelen Kim, J. (2018). Food policy councils and local governments: Creating effective collaboration for food systems change. Journal of Agriculture, Food Systems and Community Development, Volume 8, Supplement 2, pp. 11-28. https://doi.org/10.5304/jafscd.2018.08B.006**

**4. Hardesty, S., King, R., Feenstra, G., Houston, L., Joannides, J. and Lev, L. (2018). Exploring supplier-manufacturer relationships in the specialty food sector. Food Distribution Research Society Proceedings, Volume 49, Issue 1: 6-12. https://www.fdrsinc.org/wp-content/uploads/2018/03/JFDR_49.1_Full-Issue.pdf**

**5. Feenstra, G., Hardesty, S., Lev, L., Houston, L., King, R., and Joannides, J. (2017). Beyond fresh and direct: exploring the specialty food industry as a market outlet for small-and medium-sized farms. Renewable Agriculture and Food Systems 0, 1-11.** <https://doi.org/10.1017/S1742170517000722>**.**

**Selected Conferences/Workshops/Tours Organized (2019)**

**1. “UC and CSU Local Food Summit,” Helped plan and implement this cross-campus convening to identify key barriers and explore opportunities for increasing campus offerings of locally grown food, Davis, CA, June 3-4, 2019. (60 attendees).**

**2. “Uprooting Racism in the Food System: a racial equity training for California extension professionals,” co-organized this day long training with SAREP colleagues and facilitator, May 31, 2019, Cabral Agricultural Center, Stockton, CA (65 attendees).**

**3. “California Communities and Food Systems Program Team Meeting,” co-organized this half-day meeting with Christy Getz and Julia Van Soelen Kim (UCCE), May 30, 2019, Cabral Agricultural Center, Stockton, CA (20 attendees).**

**4. “31 st California Small Farm Conference,” helped organize this conference as part of the Planning Committee, February 22-23, 2019.**

**5. “Sacramento Meet the Buyer Tour,” helped organize this farmer tour to Sacramento distributors and retailers, Sacramento, CA, February 11, 2019.**

**6. “Farmer – Buyer Mixer” Oversaw, helped organize and spoke at this mixer for small and mid-scale farmers and specialty food buyers, Sebastopol Grange Hall, Sebastobpl, CA, January 8, 2019.**

**Selected Presentations (2019/ 2020)**

**1. “Food security and community gardens: Who will feed future cities?” Chair of session at “The Next Generation of Sustainable Cities” conference, UC Davis, January 24, 2020.**

**2. “Building resilience and equity into growing megalopolitan regions: engaging people**

**in regional food systems,” panel presentation at The Next Urban Giants: Building**

**Resilience and Equity into Growing Megalopolitan Regions by Greening the Urban**

**Human-Natural System workshop, July 30 – Aug 1, 2019, Seattle, WA.**

**3. “Innovations in farm-to-school in rural California,” presentation at the ASFS/AFHVS**

**Conference, June 28, 2019, Anchorage AK.**

**4. “Food policy councils as strategies for ‘Finding Home in the Wilderness’,” Panel**

**presentation at the ASFS/AFHVS Conference, June 28, 2019, Anchorage, AK.**

**5. “Engaging small farmers with the specialty food industry/ Engaging farmers in**

**values-based supply chains and food hubs,” Presentation at the ANR Small Farm**

**Workgroup meeting, Davis, CA, April 29, 2019.**

**6. “Sustainable, regional food systems,” Presentation at the Young Planners Group**

**meeting, Sacramento, CA, January 31, 2019.**

**7. “Engaging farmers in the specialty food industry,” Presentation at the Farmer-Buyer**

**Mixer, Sebastopol Grange, January 8, 2019.**

**D. Additional Information: Research Support and/or Scholastic Performance**

**Grants and Awards (selected, last 3 years)**

1. “Partnership to support California Microenterprise Home Kitchen Operations,” from Public Impact Research Initiative (PIRI). SAREP awarded $9,996.

2. “Agritourism Intensive 2020: Managing the Risks of Agritourism for California Farmers and Ranch Diversification,” from Western Extension Risk Management Education, Washington State University Extension. SAREP awarded $49,961 from 3/1/20-6/30/21.

3.“Value Chain Connections: A Regional Response to Farm to Institution Demand,” subcontracts from Community Alliance with Family Farmers (USDA LFPP grant). SAREP awarded $27,104 from 1/1/20 – 12/30/21.

4. “Riverside Unified School District Food Hub Market Feasibility Study,” subcontract from Nutrition Policy Institute, partner with RUSD on CDFA grant. SAREP awarded $21,832 from 1/1/19 – 6/15/19.

5. “Farmers Market LIFE: Using Participatory Research to Expand the Customer. Base of Farmers Market Shoppers,” subcontract from UCCE award from USDA, FMPP. SAREP awarded $119,237 from 9/30/2018 – 9/29/2021.

6. “Supporting FSMA compliance for California’s regional food hubs through training and technical assistance,” from USDA Food Safety Program. SAREP awarded $149,906 for 9/2018-8/2020.

7. “Critical Success Factors for Small and Medium-sized Farms with Direct Sales and Agritourism,” from USDA AFRI – CARE. SAREP award is $39,690 from 7/18 – 6/21.

8. “Agritourism Intensive 2018: Managing the Risks of Agritourism for California Farm and Ranch Diversification,” from Western Extension Risk Management Education. SAREP awarded $49,780 for 4/1/18-9/30/19.

9. “Increasing Regional Food Sourcing on UC Campuses by Building the Business Capacity of Food Hub Enterprises,” from UCOP Global Food Initiative. SAREP award is $84,362 from 4/27/17 -6/28/19.

10. “Growing California Agritourism Communities,” from WSARE. SAREP award is $73,010 from 4/1/17 – 9/30/19.

11. “ProCureWorks Program Evaluation,” from HealthCare Without Harm. SAREP award is $40,000 for 3/1/17 – 2/28/18.
